# Salinity Response in Chloroplasts: Insights from Gene Characterization

**DOI:** 10.3390/ijms18051011

**Published:** 2017-05-08

**Authors:** Jinwei Suo, Qi Zhao, Lisa David, Sixue Chen, Shaojun Dai

**Affiliations:** 1 Alkali Soil Natural Environmental Science Center, Northeast Forestry University, Key Laboratory of Saline-alkali Vegetation Ecology Restoration in Oil Field, Ministry of Education, Harbin 150040, China; suojinwei@nefu.edu.cn; 2 Development Center of Plant Germplasm Resources, College of Life and Environmental Sciences, Shanghai Normal University, Shanghai 200234, China; zhaoqizq@yeah.net; 3 Department of Biology, Genetics Institute, Plant Molecular and Cellular Biology Program, Interdisciplinary Center for Biotechnology Research, University of Florida, Gainesville, FL 32610, USA; lisaidavid@ufl.edu (L.D.); schen@ufl.edu (S.C.)

**Keywords:** chloroplast, gene characterization, salinity response

## Abstract

Salinity is a severe abiotic stress limiting agricultural yield and productivity. Plants have evolved various strategies to cope with salt stress. Chloroplasts are important photosynthesis organelles, which are sensitive to salinity. An understanding of molecular mechanisms in chloroplast tolerance to salinity is of great importance for genetic modification and plant breeding. Previous studies have characterized more than 53 salt-responsive genes encoding important chloroplast-localized proteins, which imply multiple vital pathways in chloroplasts in response to salt stress, such as thylakoid membrane organization, the modulation of photosystem II (PS II) activity, carbon dioxide (CO_2_) assimilation, photorespiration, reactive oxygen species (ROS) scavenging, osmotic and ion homeostasis, abscisic acid (ABA) biosynthesis and signaling, and gene expression regulation, as well as protein synthesis and turnover. This review presents an overview of salt response in chloroplasts revealed by gene characterization efforts.

## 1. Introduction

Soil salinity is one of the most severe abiotic stresses affecting agricultural yield and productivity worldwide [1]. In plants, salinity induces ion imbalance, hyperosmotic stress, oxidative damage, and other subsequent disturbances. Plants have evolved sophisticated salt-responsive signaling and metabolic processes, including photosynthetic adjustment, ion exclusion, the synthesis of compatible products, the enhancement of reactive oxygen species (ROS) scavenging, phytohormone regulation, and cell structure modulation [2].

Chloroplasts are organelles for photosynthesis. Chloroplasts also participate in many other important metabolic processes, including the biosynthesis of amino acids, vitamins, isoprenoids, fatty acids, and lipids, as well as the reduction of nitrites and sulfates [3,4]. About 3000 distinct proteins are estimated to be localized to the *Arabidopsis thaliana* chloroplasts [5,6]. Most of these proteins are encoded by the nuclear genome, and less than 150 proteins are predicted to be encoded by the plastome [7]. Previous gene characterization studies have revealed a number of genes/proteins involved in various signaling and metabolic processes in chloroplasts [7,8]. However, the characterization of salinity-responsive genes encoding chloroplast-localized proteins is limited, although many genes/proteins have been proposed to be involved in salt tolerance using transcriptomic and proteomic approaches [9,10,11,12].

Over the past twenty years, only about 53 salt-responsive genes have been characterized that encode chloroplast-localized proteins from Arabidopsis, rice (*Oryza sativa*), maize (*Zea mays*), wheat (*Triticum aestivum*), beet (*Beta vulgaris*), cotton (*Gossypium hirsutum*), mangrove (*Kandelia candel*), tobacco (*Nicotiana tabacum*), alfalfa (*Medicago sativa*), spinach (*Spinacia oleracea*), alkaligrass (*Puccinellia tenuiflora*), cowpea (*Vigna unguiculata*), pea (*Pisum sativum*), *Thellungiella halophila*, *Mesembryanthemum crystallinum*, *Porteresia coarctata*, *Suaeda salsa*, and *Synechocystis* sp. PCC 6803, respectively (Table 1). The proteins encoded by these salt-responsive genes are mainly involved in ROS scavenging, thylakoid membrane organization, photosystem II (PS II) activity, carbon dioxide (CO_2_) assimilation, photorespiration, osmotic and ion homeostasis, abscisic acid (ABA) biosynthesis and signaling, and gene expression, as well as protein synthesis and turnover (Table 1). In this review, we summarize the genes encoding the chloroplast-localized proteins in response to salinity.

## 2. Salinity-Induced Diverse ROS Scavenging Pathways in Chloroplasts

Chloroplasts are unique organelles due to their highly oxidizing metabolic activity and increased rate of electron flow, being especially prone to generating ROS, such as superoxide anion (O_2_^−^), hydrogen peroxide (H_2_O_2_), singlet oxygen (^1^O_2_), and hydroxyl radical (OH•). The presence of ROS producing centers, such as triplet chlorophylls and the electron transport chain (ETC) in PS II and PS I, make chloroplasts a major site of ROS production [13]. Salinity stress enhances ROS production, leading to severe ROS-associated damage to chloroplasts. ROS seriously disrupt normal metabolism through oxidative damage to lipids, nucleic acids, and proteins, resulting in protein destruction and the peroxidation of membrane lipids [14]. Therefore, antioxidant enzyme systems and non-enzymatic antioxidants in chloroplasts offer greater protection from oxidative damage generated from salinity stress. Several salinity-responsive genes encoding antioxidant enzymes/proteins have been cloned (Table 1), which highlight specific ROS scavenging pathways in chloroplasts under salt stress.

### 2.1. The Water-Water Cycle Detoxifies O_2_^−^ and H_2_O_2_

**Figure 1:**
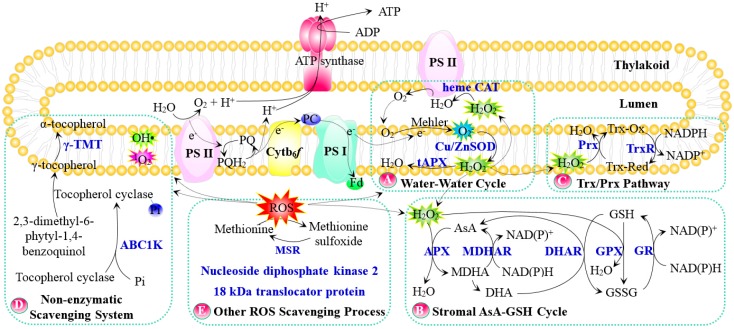
Schematic presentation of ROS scavenging pathway in chloroplasts. (**A**) Water–Water cycle; (**B**) Stromal AsA–GSH cycle; (**C**) Trx/Prx pathway; (**D**) Non-enzymatic scavenging system; (**E**) Other ROS scavenging process. The solid line indicates a single-step reaction, and a dotted line indicates the movement of molecules. Substrates and products are in black font, proteins are in blue bold font, and P in a blue circle indicates phosphorylated protein. Abbreviations: ABC1K, activity of bc1 complex-like kinase; APX/tAPX, ascorbate peroxidase/thylakoid ascorbate peroxidase; AsA, ascorbate; Cu/Zn SOD, copper/zinc superoxide dismutase; Cytb_6_*f*, cytochrome b_6_*f* complex; DHA, dehydroascorbate; DHAR, dehydroascorbate reductase; Fd, Ferredoxin; GPX, glutathione peroxidase; GR, glutathione reductase; GSH, reduced glutathione; GSSG, oxidized glutathione; H_2_O_2_, hydrogen peroxide; hemeCAT, heme catalase; MDHA, monodehydroascorbate; MDHAR, monodehydroascorbate reductase; MSR, sulfoxide reductase; ^1^O_2_, singlet oxygen; O_2_, oxygen; O_2_^−^, superoxide anion; OH•, hydroxyl radical; PC, plastocyanin; Prx, Trx-dependent peroxidase; PS II, photosystem II; ROS, reactive oxygen species; γ-TMT, γ-tocopherol methyltransferase; Trx-Ox, oxidized thioredoxin; Trx-Red, reduced thioredoxin; TrxR, thioredoxin reductase.

The water-water cycle operates as a ROS scavenging pathway in chloroplasts, which is essential for salinity tolerance (Figure 1A). Oxygen (O_2_) generated in chloroplasts during photosynthesis can accept electrons passing through PS II and PS I, resulting in the formation of O_2_^−^ by the Mehler reaction [15]. The major site of O_2_^−^ production is the thylakoid membrane-bound primary electron acceptor of PS I. It has been revealed that the acceptor side of the ETC in PS II also provides sides (Q_A_, Q_B_) with electron leakage to O_2_ producing O_2_^−^. Once produced on the internal “lumen” membrane surface, O_2_^−^ may be protonated to HO_2_^−^, which initiates lipid peroxidation. On the “stromal” membrane surface, a membrane attached copper/zinc superoxide dismutase (Cu/Zn SOD) in the vicinity of PS I catalyzes the dismutation of O_2_^−^ into O_2_ and H_2_O_2_, and then the generated H_2_O_2_ is reduced to H_2_O by a membrane-bound thylakoid ascorbate peroxidase (tAPX), which is the commonly named water–water cycle [13].

The overexpression of *Cu/Zn SOD* in chloroplasts of Arabidopsis [16], tobacco [17,18], Chinese cabbage (*Brassica campestris* L. ssp. *pekinensis* cv. Tropical Pride) [19], and cotton [20] can enhance salinity tolerance through reducing ROS (Table 1). Similarly, tobacco plants with an overexpression of *APX* in chloroplasts showed a higher resistance to salt stress, and the isolated chloroplasts from the transgenic lines also showed higher APX activity than wild-type control plants [21]. These results indicated that the thylakoidal scavenging system of ROS is essential for salt tolerance.

Although catalase (CAT) has not been found in chloroplast stroma, PS II membranes associate with a heme CAT [22]. The CAT does not directly participate in the water–water cycle, but protects water oxidase in the lumen if the water–water cycle does not operate properly and H_2_O_2_ diffuses to the lumen [15]. An increased defense against oxidative damage induced by salt stress was conferred by targeting CAT to chloroplasts in both Chinese cabbage [19] and cotton plants [20].

### 2.2. Stromal Ascorbate (AsA)-Glutathione (GSH) Cycle

Salinity-induced ROS generated in thylakoids and/or stroma undergo detoxification by the stromal AsA–GSH cycle. In this cycle, H_2_O_2_ is reduced to H_2_O catalyzed by stroma APX using AsA as the electron donor, and the oxidized AsA can be reduced back to AsA by monodehydroascorbate reductase (MDHAR), or be converted into dehydroascorbate (DHA) spontaneously. Then, DHA is reduced to AsA by dehydroascorbate reductase (DHAR) at the expense of GSH, generating oxidized glutathione (GSSG). Furthermore, GSSG is reduced by glutathione reductase (GR) using NADPH as an electron donor [14]. Genes encoding the aforementioned enzymes have been reported to be regulated by salinity (Figure 1B, Table 1). Tobacco plants overexpressing genes of *MDHAR* [23] and *DHAR* [24] showed significantly high enzyme activities of MDHAR and DHAR, as well as an increased level of reduced AsA and improved survival under salt stress. In addition, *OsGR3* was markedly induced in rice under salt treatment [25], and the salinity sensitivity of rice was increased when the *OsGR3* gene was knocked out [26]. These results indicate that stromal ROS scavenging in chloroplasts is crucial for redox homeostasis and supplying NADP^+^, leading to the reduced loading of the ETC. Overall, this contributes to enhancing a plant’s ability to withstand adverse environmental conditions [13].

### 2.3. Thioredoxin/Peroxiredoxin (Trx/Prx) and Glutathione Peroxidase (GPX) Pathway

Salinity-induced H_2_O_2_ is a potent oxidant for protein thiol groups, which are highly susceptible to oxidation. The thiol reduction is mainly controlled by the Trx/Prx pathway and the GPX pathway. Trx acts as an electron donor that couples with Trx-dependent peroxidase (Prx) to scavenge H_2_O_2_, and thioredoxin reductase (TrxR) utilizes NADPH to keep the Trx/Prx system in a reduced state (Figure 1C, Table 1) [65]. In this process, electrons are taken from NADPH via TrxR, and then transferred to the active site of Trx, which can reduce protein disulfides or other substrates. Transgenic Arabidopsis overexpressing a *S. salsa* thylakoid membrane-attached *SsPrxQ* gene showed enhanced salt tolerance [27]. Similarly, a NADPH thioredoxin reductase (OsNTRC) gene was cloned from rice, which encodes a chloroplast-localized bifunctional enzyme with both TrxR and thioredoxin activity [28]. An Arabidopsis *NTRC* knockout mutant showed growth inhibition and hypersensitivity to salt stress [28]. In addition, GPX is also involved in the reduction of H_2_O_2_, using GSH as the electron donor. Overexpressing wheat *W69* and *W106* genes, which encode chloroplast GPXs, can improve salt and H_2_O_2_ tolerance in Arabidopsis [29]. These results provided evidence that the Trx/Prx system and GPX pathway in chloroplasts are important for the stroma H_2_O_2_ removal in salt-stressed plants.

### 2.4. Non-Enzymatic OH• and ^1^O_2_ Scavenging System

Salinity-induced O_2_^−^ generation may trigger more reactive OH• formation through the Fenton reaction. In PS II, O_2_ of the ground (triplet) state is excited to ^1^O_2_ by the reaction center chlorophyll of the triplet excited state [15]. Primarily, OH• and ^1^O_2_ are scavenged by AsA, GSH, and tocopherol in chloroplasts [13].

Among these OH• and ^1^O_2_ scavengers, tocopherol is a thylakoid membrane-localized lipid antioxidant, which can protect photosynthetic membranes from oxidative damage by scavenging ROS and prevent the propagation of lipid peroxidation under stress conditions [66]. Tocopherol biosynthesis is a finely balanced process in chloroplasts. Arabidopsis chloroplast plastoglobule-localized ABC1 (for activity of bc1 complex)-like kinase ABC1K3 phosphorylates tocopherol cyclase, possibly stabilizing it at plastoglobules and regulating tocopherol biosynthesis [67]. In addition, *AtSIA1* encodes a chloroplast-localized ABC1-like kinase, which is salinity-induced in Arabidopsis. Transgenic Arabidopsis seedlings that overexpress *AtSIA1* showed a higher tolerance to salt stress than Col-0 and the *AtSIA1* knockout mutant [30]. Additionally, γ-tocopherol methyltransferase (γ-TMT) is another important enzyme regulating tocopherol synthesis (Figure 1D, Table 1). Under NaCl stress, the overexpression of Arabidopsis *γ-TMT* in tobacco chloroplasts converted more γ-tocopherol to α-tocopherol, enhanced sugar transport, and reduced ROS contents and ion leakage, which ultimately contributed to salt stress alleviation [31].

### 2.5. Other Genes Involved in Chloroplast ROS Scavenging

Several genes have been proposed to regulate the ROS scavenging process (Figure 1E, Table 1). Rice *WSL12* encodes a chloroplast nucleoside diphosphate kinase 2 (NDPK2), which plays an important role in chloroplast development and chlorophyll biosynthesis by regulating multiple gene expression levels [32]. The *WSL12* mutant showed high O_2_^−^ levels and sensitivity to salinity [32], probably due to the association of NDPK2 with ROS signaling and oxidative stress. Overexpressing *NDPK2* in Arabidopsis chloroplasts induces a higher expression of multiple antioxidant genes (e.g., *peroxidase*, *CAT*, *Trx*, *TrxR*, and *Prx*) [68]. Similarly, transgenic sweet potato (*Ipomoea batatas*) with an expression of *AtNDPK2* in chloroplasts showed an enhanced tolerance to salinity and increased activities of peroxidase, APX, and CAT [33].

As expected, the concentrations of photoreactive tetrapyrrole intermediates are tightly controlled as they can generate ROS under a variety of environmental stimuli. The Arabidopsis genome contains a single 18 kDa translocator protein (TSPO)-encoding gene *AtTSPO*, which is normally localized to the endoplasmic reticulum and vesicles, and is translocated to chloroplasts in the presence of 150 mM NaCl. *AtTSPO* is involved in transporting tetrapyrrole intermediates and protecting chloroplasts from oxidative damage [34].

In addition, methionine sulfoxide reductase (MSR) plays a role in the plant oxidative stress response. The methionine (Met) residue in this protein is especially sensitive to oxidation, leading to the formation of S- and R-epimers of methionine sulfoxide. The sulfoxide, in turn, can be enzymatically reduced back to Met by MSR. Thus, the oxidation and the enzymatically catalyzed reduction of Met is probably a critical molecular mechanism for cellular redox regulation under stress conditions. Transgenic rice plants overexpressing the chloroplast-localized *OsMSRA4.1* showed enhanced viability during salt stress, implying that MSR is important for Met reduction in chloroplasts [35].

## 3. Thylakoid Membrane Organization and Photosynthesis

Plants’ photosynthetic machinery respond to salt stress by regulating thylakoid membrane fluidity and remodeling membrane lipid composition, thus maintaining an environment suitable for the function of critical integral proteins during stress [69].

**Figure 2:**
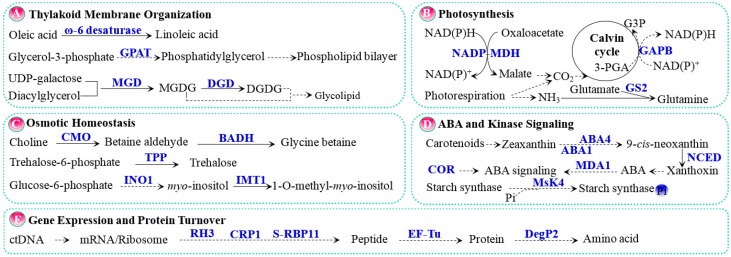
Salt tolerance pathways in chloroplasts. (**A**) Thylakoid membrane organization; (**B)** Photosynthesis; (**C**) Osmotic homeostasis; (**D**) ABA and kinase signaling; (**E**) Gene expression and protein turnover. The solid line indicates a single-step reaction, and the dashed line indicates a multistep reaction. Substrates and products are in black font, proteins are in blue bold font, and P in a blue circle indicates phosphorylated protein. Abbreviations: 3-PGA, glycerate-3-phosphate; ABA, abscisic acid; ABA1, zeaxanthin epoxidase; ABA4, neoxanthin synthase; BADH, betaine aldehyde dehydrogenase; CMO, choline monooxygenase; CO_2_, carbon dioxide; COR, cold-responsive protein; CRP1/S-RBP11, RNA-recognition motif containing protein; DegP2, prokaryotic trypsin-type Deg/Htr serine protease; DGD, digalactodiacylglycerol synthase; DGDG, digalactodiacylglycerol; EF-Tu, translation elongation factor; G3P, glyceraldehyde-3-phosphate; GAPB, glyceraldehyde 3-phosphate dehydrogenase beta subunit; GPAT, glycerol-3-phosphate acyltransferase; GS2, glutamine synthetase; IMTI, inositol methyl transferase; INO1, l-*myo*-inositol 1-phosphate synthase; MDA1, transcription termination factor; MGD, monogalactosyldiacylglycerol synthase; MGDG, monogalactosyldiacylglycerol; MsK4, glycogen synthase kinase 3 like kinase; NADP-MDH, NADP^+^-dependent malate dehydrogenase; NCED, 9-*cis*-epoxycarotenoid dioxygenase; RH3, DEAD-box RNA helicase; TPP, trehalose-6-phosphate phosphatase.

The modification of membrane fluidity is mediated by the changes in unsaturated fatty acid levels of thylakoid membranes. *Fad6* encodes a chloroplast-localized ω-6 desaturase, which is a fatty acid desaturase that catalyzes the conversion of oleic acid (18:1) to linoleic acid (18:2) by inserting a double bond at the ω-6 position. The Arabidopsis *fad6* mutant has an increased sensitivity to salt stress, implying that Fad6 is required for salt tolerance during early seedling development [36]. In addition, a chloroplast-localized glycerol-3-phosphate acyltransferase (GPAT) is one of the main factors that determine the content of *cis*-unsaturated fatty acids in the phosphatidylglycerol of thylakoid membranes (Figure 2A, Table 1) [37]. The overexpression of *GPAT* in tomato increased the *cis*-unsaturated fatty acid content of thylakoid membranes, and the transgenic plants exhibited higher activities of chloroplastic antioxidant enzymes, lower ROS contents, and a better photosynthetic performance, as well as an increased efficiency in alleviating PS II photoinhibition [37]. The increased unsaturation of fatty acids seems to enhance structural flexibility to the thylakoid membranes, which is favorable to thylakoid membrane binding to antioxidant enzymes for excess ROS scavenging [37]. Importantly, the unsaturation of fatty acids might boost the tolerance of PS II to salt stress by accelerating the repair of photodamaged D1. The PS II reaction center subunits D1 and D2 are enclosed by a belt of 11 lipids, which provides a flexible environment and fosters a high mobility of subunits. This would be beneficial to the degradation of damaged D1, the acceleration of de novo synthesis, and the insertion of the D1 protein [70]. However, the specific steps of the PS II repair that are regulated by the unsaturation of fatty acids still remain to be clarified.

Two galactolipids, monogalactosyldiacylglycerol (MGDG) and digalactodiacylglycerol (DGDG), are major constituents of photosynthetic membranes in chloroplasts (Figure 2A, Table 1) [71]. MGDG has a conical shape with nonbilayer-forming characteristics, providing a high lateral pressure on the proteins embedded in the membrane. This permits the dense packing of membrane proteins, which facilitates the maintenance of the stability of the membrane structure. Furthermore, the non-bilayer lipid can easily segregate from the membrane and is crucial for the self-regulation of lipid content in the thylakoid membrane through the formation of osmiophilic lipid droplets in the chloroplasts. In addition, DGDG, a bilayer-prone lipid, is involved in lipid-mediated contact between adjacent trimers of the light harvesting complex II [71]. This is important for the stability of the lamellar structure of chloroplast membranes. Crystallization studies have revealed that MGDG was associated with the core of the reaction center of PS I, PS II, and the cytochrome b_6_*f* complex, while DGDG was a component of PS I, PS II, and the light-harvesting complex of PS II [38,72,73]. Thus, they can provide a membrane environment to physically support the photosynthetic complex, and also contribute directly to various photosynthesis-related processes. The biosynthesis of the two galactoglycerolipids is catalyzed by MGDG synthase (MGD) and DGDG synthase, respectively [74]. A chloroplast outer envelope membrane-localized MGD encoding gene has been cloned in rice, and transgenic tobacco plants that overexpress *OsMGD* exhibited significantly higher levels of MGDG and DGDG, as well as higher DGDG/MGDG ratios than wild-type plants. Chloroplasts from salt-stressed *OsMGD* transgenic tobacco had well-developed thylakoid membranes and properly stacked grana lamellae, whereas the chloroplasts from salt-stressed wild-type plants were fairly disorganized and had large membrane-free areas [38]. Therefore, the increased levels of MGDG and DGDG could contribute to the organization of the plant photosynthetic membrane structure for the enhancement of salt resistance.

Some chloroplast-localized proteins are critical for the maintenance of PS II activity in response to salt stress. Rubredoxin (RUB) is a small, non-heme protein, which attaches to the thylakoid membranes and is exposed to the stroma, acting as an electron carrier in a variety of biochemical processes, such as the detoxification of ROS [75]. Mutant analysis of the *RUB* gene in the green alga *Chlamydomonas reinhardtii* suggests that RUB may be necessary for normal PS II activity in a diverse set of organisms that perform oxygenic photosynthesis. Knockout mutants of *RUB* orthologs in the cyanobacterium *S.* sp. PCC 6803 and Arabidopsis also imply that the activity and stability of PS II are specifically affected [76]. In addition, the overexpression of the *PutRUB* gene from alkaligrass in Arabidopsis increased the tolerance to NaCl and NaHCO_3_ stress. This was probably due to decreasing H_2_O_2_ accumulation for chloroplast redox balance [39]. Similarly, a *RCI* gene encoding a chloroplast membrane protein has also been reported to be involved in the regulation of PS II activity. RCI is a homolog of a plasma membrane protein 3 family and contains two putative transmembrane domains. Arabidopsis plants overexpressing the wheat *RCI* performed better than the wild-type under salinity stress. Transgenic plants showed significantly higher PS II activity in terms of the maximum photochemical efficiency, and higher proline and chlorophyll contents than wild-type plants [40].

Under salt stress, the maintenance of stable CO_2_ assimilation would be important to salinity tolerance. NADP^+^-dependent malate dehydrogenase (NADP-MDH) is responsible for the reduction of oxaloacetate (OAA) to malate in chloroplasts, which was suggested to be crucial for CO_2_ fixation (Figure 2B, Table 1). In C3 plants, NADP-MDH is essential for the balance of reducing equivalents between chloroplasts and cytoplasm via the malate/oxaloacetate shuttle. In C4 plants, NADP-MDH is located exclusively in the mesophyll chloroplasts for C4-photosynthesis. *NADP-MDH* transcripts in the facultative Crassulacean acid metabolism (CAM) plant *M. crystallinum* significantly accumulated in response to salt stress. The salinity-induced expression of *NADP-MDH* suggests that CO_2_ fixation is enhanced, which implies that the facultative, halophytic *M. crystallinum* shifts the photosynthesis carbon fixation mode from a C3 to a CAM in response to salinity [41]. In addition, the glyceraldehyde 3-phosphate dehydrogenase beta subunit (GAPB) is a key enzyme for the conversion of glycerate-3-phosphate (3-PGA) to glyceraldehyde-3-phosphate interacting with ATP and NADPH. 3-PGA can accept electrons from NADPH, preventing the ROS-induced deceleration of PS II repair (Figure 2B, Table 1). Arabidopsis overexpressing *ThGAPB* exhibited higher recycling rates of ADP and NADP^+^. This would reduce ROS production, contributing to the maintenance of photosynthetic efficiency under salinity conditions [42].

Additionally, salt stress-induced stomatal closure limits the CO_2_ concentration in cells, which causes the over-reduction of photosynthetic ETC. Under such a condition, salinity-responsive ribulose-1, 5-bisphosphate carboxylase/oxygenase (Rubisco) operates as an oxygenase, and photorespiration is activated in response to salt stress. Phosphoglycerate and CO_2_ generated from photorespiration enter the Calvin cycle, and the consumption of NADPH and ATP may contribute to the dissipation of excess light energy or reducing power, for preventing the over-reduction of ETC. Interestingly, transgenic rice overexpressing a chloroplastic glutamine synthetase encoding gene *GS2* increased their photorespiration capacity for improving salt tolerance (Figure 2B, Table 1) [43]. 

## 4. Osmotic and Ion Homeostasis

To withstand salinity-induced osmotic stress, plants synthesize and accumulate compatible solutes/osmoprotectants for stabilizing proteins, membranes, and even transcriptional and translational machineries in the cells. Major osmoprotectants include betaines, amino acids (e.g., proline), non-reducing sugars (e.g., trehalose and arabitol), and polyols [77]. Betaine is a quaternary ammonium compound found in a wide variety of plants, animals, and microorganisms [78]. Salinity-induced betaine accumulates in the chloroplasts of many halotolerant plants, stabilizing the quaternary structure of enzymes and protein complexes, as well as the highly-ordered structure of membranes in photosynthetic machinery [79]. For example, it can stabilize the Rubisco and PS II oxygen-evolving complex and accelerate the repair of photodamaged PS II under salt or other abiotic stress [80,81]. In plants, betaine is synthesized by several betaine-biosynthetic enzymes, such as choline monooxygenase (CMO) and betaine aldehyde dehydrogenase (BADH) (Figure 2C, Table 1). Genes encoding CMO and BADH have been cloned and targeted to the chloroplast genome of various plant species (Table 1). In transgenic plants, the enzyme activities and betaine levels were increased, leading to an improved photosynthetic performance and enhanced tolerance to salt stress [44,45,46,47]. Importantly, trehalose and inositol also act as important osmolytes in chloroplasts, which can significantly enhance the salt tolerance of the transgenic plants. Chloroplast-localized trehalose-6-phosphate phosphatase (TPP) catabolizes trehalose-6-phosphate to generate trehalose, which is the final step of trehalose metabolism (Figure 2C, Table 1). Arabidopsis plants deficient in *AtTPPD* were hypersensitive, whereas plants overexpressing *AtTPPD* were tolerant to high salinity [48]. In addition, the co-expression of *PcINO1* and *McIMT1* in chloroplasts allowed the transgenic tobacco plants to perform better in terms of growth potential and photosynthesis rates with increased levels of *myo*-inositol and methylated inositol under salt stress [49].

Besides osmotic balance, ion homeostasis is also vital for plant cells to cope with salinity. Extra Na^+^ in chloroplasts destroys the thylakoid membrane structure, causing the inactivation of PS II and PS I and inhibiting the repair of photodamaged PS II, as well as decreasing photosynthesis electron transport, and therefore, the Na^+^ concentration in chloroplasts must be well-controlled. *AtCHX23* encodes a putative Na^+^(K^+^)/H^+^ exchanger, which is localized in the chloroplast envelope and functions by putatively regulating the homeostasis of the cytoplasmic and stromal pH and Na^+^ concentration through the sequestering of Na^+^ entering into chloroplasts. Arabidopsis *CHX23* mutants displayed a high sensitivity to NaCl [50]. Moreover, *NHD1* encodes a sodium hydrogen antiporter, which is localized to the chloroplast envelope. Arabidopsis NHD1 functions as a chloroplast sodium exporter, protecting chloroplasts from deleterious Na^+^ accumulation after salt exposure. *NHD1* T-DNA insertion mutants showed high Na^+^ levels in chloroplasts, resulting in a markedly impaired photosynthetic performance as revealed by a lower quantum yield of PS II and increased non-photochemical quenching [51]. In addition, several members of the putative K^+^-efflux antiporters (KEA), such as KEA1, KEA2, and KEA3, function as chloroplast K^+^/H^+^ antiporters for regulating the osmotic pressure, pH, and ion homeostasis of chloroplasts. KEA1 and KEA2 are chloroplast inner envelope membrane proteins, and function to release K^+^ from the chloroplasts in exchange for H^+^ influx, whereas KEA3 is a thylakoid membrane protein that uptakes K^+^ into the thylakoid lumen. Arabidopsis loss-of-function mutants of these three genes have been analyzed, and the higher-order mutants showed increasingly impaired photosynthesis, along with altered chloroplast pH homeostasis [52].

In addition, a chloroplast stroma-localized YL1 protein is an YqeH-type GTPase, involved in the regulation of Na^+^ delivery in response to salt stress. The expression of Arabidopsis *YL1* was markedly reduced under high salinity. Shoots of the *YL1* mutant accumulated significantly higher levels of Na^+^ than wild type under salt stress. The expression of *Abscisic acid insensitive 4 (ABI4)* was increased and *high-affinity K^+^ transporter 1 (HKT1)* was suppressed in the mutant shoots. *HKT1*, encoding a K^+^/Na^+^ symporter, is an important regulator that can directly retrieve Na^+^ from the xylem sap back to the phloem of the shoot and unload it in the root for ion homeostasis [82]. ABI4 has recently been reported to act as a negative regulator that could directly bind to the promoter region and inhibit *HKT1* expression in Arabidopsis [83]. It is speculated that the reduction of *YL1* and the induction of *ABI4* may be an adaptive mechanism to achieve Na^+^ equilibrium in the entire plant, which needs to be further investigated [53].

## 5. ABA and Kinase Signaling Pathways

The investigation of cellular ABA level fluctuation, ABA perception, and ABA-mediated signaling in response to salinity is pivotal for understanding plant stress tolerance. All except the last two steps of ABA de novo biosynthesis occur in plastids [84]. Most of the genes involved in ABA biosynthesis have been identified, among which, *ABA1*, *ABA4*, and *NCED* encode plastid-localized zeaxanthin epoxidase, neoxanthin synthase, and 9-*cis*-epoxycarotenoid dioxygenase, respectively (Figure 2D, Table 1) [54,55,85,86]. Salt stress has some influence on the cellular ABA content, and it was reported that *AtABA1* and *VuNCED1* were up-regulated by salt stress [54,55].

In addition, *MDA1* encoding a transcription termination factor family protein, is likely required for a proper ABA response in chloroplasts. An Arabidopsis *MDA1* mutant showed reduced sensitivity to ABA, enhanced salt tolerance, and some salt-responsive gene expression [56]. *MDA1* deficit in plants may disrupt chloroplast homeostasis and negatively affect ABA retrograde signaling, which elicits the nuclear-encoded functions required for coping with salt stress [56]. Moreover, a gene encoding stroma-localized cold-responsive (COR) protein, *COR15B*, was up-regulated under salt stress in an ABA-dependent manner. Therefore, *COR15B* was proposed to be a potential member in the ABA signaling regulatory network in the Arabidopsis response to salt stress [57].

Protein kinases are major components of intracellular signal transduction, which mediate various signaling pathways that enable plant cells to rapidly acclimate and prevail in challenging environmental conditions. MsK4 is a plastid-localized novel alfalfa glycogen synthase kinase 3 like kinase, which was reported to be a novel signaling component in the regulation of starch metabolism and the salt stress response. Starch metabolism is highly sensitive to environmental changes and its accurate regulation is crucial for the adaptation of carbon and energy flow in response to stress conditions. Fluxes of carbon into and out of starch are extremely complex and must be highly controlled. Evidence is emerging that reversible protein phosphorylation is crucial for the regulation of starch-associated carbon metabolism, and the phosphorylation of starch metabolic enzymes has been shown to control their activities and protein complex formation. It has been found that MsK4 can bind to some isoforms of soluble and granule-bound starch synthases in vitro, suggesting that the enzymes involved in starch metabolism might be direct targets of the MsK4 action (Figure 2D, Table 1). High-salinity induced the activity of MsK4 kinase in alfalfa and Arabidopsis. Moreover, *MsK4* overexpressing transgenic plants showed an enhanced salt tolerance and significantly increased starch content. This opens new perspectives as to how metabolic carbon flux can be regulated in response to salinity, and provides links between stress signaling and metabolic adaptation [58].

## 6. Chloroplast Gene Expression and Protein Turnover

The salt-responsive gene expression of the chloroplast genome must be very precisely controlled, and the targeting of nuclear-encoded proteins into the chloroplasts is important in regulating chloroplast gene expression. Chloroplast gene expression is mainly regulated at the level of posttranscriptional RNA metabolism, including RNA processing, editing, splicing, decay, and translational control [87]. The functions of most RNA molecules rely on a well-defined three-dimensional structure, and the correct folding of RNA molecules requires the assistance of diverse RNA binding proteins (RBPs) [88]. A variety of nuclear-encoded RBPs are targeted to chloroplasts and play indispensable roles in the plant salt response through the posttranscriptional regulation of RNA metabolism and gene expression in chloroplasts, such as DEAD-box RNA helicases (RHs) and RNA-recognition motifs (RRMs)-containing proteins (Figure 2E, Table 1). Arabidopsis chloroplast-localized RH3 has been demonstrated to function in intron splicing and ribosome biogenesis. *RH3* mutants displayed more sensitivity to salt stress and the splicing of several intron-containing chloroplast genes was inhibited [59]. Besides, Arabidopsis CRP1 and S-RBP11, two of the chloroplast-localized RRM containing proteins, were also shown to be involved in the salt stress response. Seed germination of the *CRP1* mutant was delayed compared with that of the wild-type seeds under salt stress [60]. Similarly, transgenic Arabidopsis overexpressing *S-RBP11* showed an increased salt tolerance, whereas *S-RBP11* mutants were shown to be more sensitive to salt stress [61].

Moreover, protein synthesis, processing, and degradation in chloroplasts are also important for plant salt stress adaption (Figure 2E, Table 1). The expression of pea *TufA*, which encodes a chloroplast translation elongation factor (EF-Tu), was down-regulated in response to salinity [62]. EF-Tu is an essential component for polypeptide elongation during protein synthesis. It is reported that EF-Tu might have a chaperone-like property of refolding the denatured proteins or preventing their aggregation under heat stress [89].

Salt-induced excess excitation energy may cause the photodamage of PS II. The D1 protein is one of the core proteins in the PS II reaction center, which is the main target of oxidative damage. The de novo synthesis, assembly, and rapid degradation of the D1 protein are necessary for the efficient PS II repair in salt-stressed plants [63,90]. In cyanobacterium *S.* sp. PCC 6803, the transcription and translation of the D1 protein encoding gene *psbA* was decreased, and the repair of the photodamaged D1 protein was also inhibited under salt stress [63]. Moreover, a prokaryotic trypsin-type Deg/Htr serine protease (DegP2) was found to be vital for the photodamaged D1 protein degradation under salt stress [91]. DegP2 is peripherally associated with the outer surface of the thylakoid membrane, and involved in the primary cleavage of the photodamaged D1 protein on the stromal DE loop. The expression of Arabidopsis *DegP2* was down-regulated under high salt stress (400 mM NaCl), while the amount of DegP2 protein was increased significantly under stress [64]. This would induce its molecular chaperone and proteolytic activities to enhance the repair of oxygen-evolving PS II in salinity-stressed plants.

## 7. Conclusions

Chloroplasts have evolved fine-tuned pathways for the salt response. In this paper, we reviewed and discussed the salt-responsive genes encoding chloroplast-localized proteins, which represent several crucial pathways in chloroplasts in response to salinity, such as chloroplast ROS scavenging (water–water cycle, stromal AsA-GSH cycle, Trx/Prx pathway, and non-enzymatic scavenging system), photosynthetic thylakoid membrane modulation, CO_2_ assimilation, the synthesis of osmoprotectant, and ion homeostasis regulation, as well as chloroplast gene expression and protein turnover. These provide important molecular information for better understanding the chloroplast salt response, and allow us to expand our knowledge on the adaptation of this photosynthetic apparatus to salinity stress. However, the photosynthetic machinery and chloroplast metabolic pathways for salt tolerance are too complicated to be interpreted by the genes characterized so far. Although a large number of candidate genes/proteins have been identified in chloroplasts from plants under salt treatments using large-scale genomic, transcriptomic, and proteomic approaches [9,10,11,12,92], further investigations of their biological functions in salinity tolerance are needed. More importantly, a deeper analysis of the post-translational modifications and protein-protein interactions of these salt-responsive proteins will facilitate a thorough understanding of the complicated salt-responsive networks in chloroplasts.

## Figures and Tables

**Table 1:** List of genes encoding chloroplast proteins in response to salinity.

Gene Name	Plant Species	Encoding Protein	Salt Treatment Condition	Ref.
Stress and defense (22)				
*Cu/Zn SOD*	Alkaligrass (*Puccinellia tenuiflora*)	Copper/zinc superoxide dismutase	NaCl (0, 50, 100 mM; 21 days); NaHCO_3_ (0, 3, 5 mM; 21 days)	[16]
*Cu/Zn SOD*	Rice (*Oryza sativa*)	Copper/zinc superoxide dismutase	NaCl (300 mM; 2, 4, 6, 8, 10 days)	[17]
*Cu/Zn SOD*	Mangrove (*Kandelia candel*)	Copper/zinc superoxide dismutase	NaCl (100, 300 mM; 8, 24 h; 1, 2, 3 weeks)	[18]
*Cu/Zn SOD*	Maize (*Zea mays*)	Copper/zinc superoxide dismutase	NaCl (0, 50, 100, 150, 200 mM; 10 days; 4 weeks)	[19]
*Cu/Zn SOD*	Cotton (*Gossypium hirsutum*)	Copper/zinc superoxide dismutase	NaCl (50, 100, 150, 200 mM; 1, 2, 3, 4 weeks)	[20]
*APX*	*Arabidopsis thaliana*	Ascorbate peroxidase	NaCl (300 mM; 8 days)	[21]
*CAT*	Maize (*Z. mays*)	Catalase	NaCl (0, 50, 100, 150, 200 mM; 10 days; 4 weeks)	[19]
*CAT*	Cotton (*G. hirsutum*)	Catalase	NaCl (50, 100, 150, 200 mM; 1, 2, 3, 4 weeks)	[20]
*MDHAR*	Mangrove (*Avicennia marina*)	Monodehydroascorbate reductase	NaCl (200 mM; 4 days)	[23]
*DHAR*	Rice (*O. sativa*)	Dehydroascorbate reductase	NaCl (100, 150, 200 mM; 12, 14 days)	[24]
*GR3*	Rice (*O. sativa*)	Glutathione reductase	NaCl (200 mM; 0, 4, 8, 12, 16, 20, 24 days)	[25]
*GR3*	Rice (*O. sativa*)	Glutathione reductase	NaCl (100 mM; 7, 15 days)	[26]
*PrxQ*	*Suaeda salsa*	Peroxiredoxin Q	NaCl (0, 100, 150 mM; 3 weeks)	[27]
*NTRC*	Rice (*O. sativa*)	NADPH thioredoxin reductase	NaCl (170 mM; 1, 3 days)	[28]
*W69*	Wheat (*Triticum aestivum*)	Glutathione peroxidase	NaCl (150 mM; 7 days)	[29]
*W106*	Wheat (*T. aestivum*)	Glutathione peroxidase	NaCl (150 mM; 7 days)	[29]
*SIA1*	Arabidopsis	ABC1-like kinase	NaCl (200 mM; 3 days)	[30]
*γ-TMT*	Arabidopsis	γ-tocopherol methyltransferase	NaCl (0, 200, 300, 400 mM; 12, 24, 48 h; 4 weeks)	[31]
*WSL12*	Rice (*O. sativa*)	Nucleoside diphosphate kinase	NaCl (100, 150, 200 mM; 0, 1, 1.5, 2, 2.5, 3, 3.5, 4, 4.5, 5, 5.5, 6, 6.5 days)	[32]
*NDPK2*	Arabidopsis	Nucleoside diphosphate kinase 2	NaCl (200 mM; 14 days)	[33]
*TSPO*	Arabidopsis	18 kDa translocator protein	NaCl (150 mM; 1, 3, 6, 12, 24 h)	[34]
*MSRA4.1*	Rice (*O. sativa*)	Methionine sulfoxide reductase	NaCl (100 mM; 2 days)	[35]
Thylakoid membrane organization and PSII activity (5)				
*Fad6*	Arabidopsis	ω-6 desaturase	NaCl (0, 75, 100, 125 mM; 8 days); (300 mM; 0, 1, 3, 6, 12, 24 h)	[36]
*GPAT*	Tomato (*Lycopersicon esculentum*)	Glycerol-3-phosphate acyltransferase	NaCl (200 mM; 1, 3, 5, 7 days); (150 mM; 30 days)	[37]
*MGD*	Rice (*O. sativa*)	Monogalactosyl-diacylglycerol synthase	NaCl (0, 200 mM; 10 days)	[38]
*RUB*	Alkaligrass (*P. tenuiflora*)	Rubredoxin family protein	NaCl (100, 125 mM; 10 days); NaHCO_3_ (1.5, 3 mM; 10 days)	[39]
*RCI*	Wheat (*T. aestivum*)	Rare cold inducible protein	NaCl (150 mM; 2 weeks)	[40]
Photosynthesis and photorespiration (3)				
*NADP-MDH*	*Mesembryanthemum crystallinum*	NADP^+^-dependent malate dehydrogenase	NaCl (400 mM; 1, 6, 12, 30, 72, 126 h)	[41]
*GAPB*	*Thellungiella halophila*	Glyceraldehyde 3 phosphate dehydrogenase β subunit	NaCl (200 mM; 2 weeks)	[42]
*GS2*	Rice (*O. sativa*)	Glutamine synthetase	NaCl (150 mM, 12 days)	[43]
Osmotic and ion homoestasis (12)				
*CMO*	Spinach (*Spinacia oleracea*)	Choline monooxygenase	NaCl (50, 100 mM; 0, 3, 6, 9, 12, 15 weeks)	[44]
*CMO*	Beet (*Beta vulgaris*)	Choline monooxygenase	NaCl (0, 100, 150 mM; 36 days)	[45]
*BADH*	Spinach (*S. oleracea*)	Betaine aldehyde dehydrogenase	NaCl (50, 100 mM; 0, 3, 6, 9, 12, 15 weeks)	[44]
*BADH*	Tobacco (*Nicotiana tabacum*)	Betaine aldehyde dehydrogenase	NaCl (100, 200, 300, 400, 500 mM; 1 month)	[46]
*BADH*	Spinach (*S. oleracea*)	Betaine aldehyde dehydrogenase	NaCl (0, 75, 150 mM; 3 weeks)	[47]
*TPPD*	Arabidopsis	Trehalose-6-phosphate phosphatase	NaCl (200 mM; 0, 1, 3, 8 h)	[48]
*INO1*	*Porteresia coarctata*	l-*myo*-inositol 1-phosphate synthase	NaCl (100, 200, 300, 400 mM; 96 h)	[49]
*IMT1*	*M. crystallinum*	Inositol methyl transferase	NaCl (100, 200, 300, 400 mM; 96 h)	[49]
*CHX23*	Arabidopsis	Na^+^(K^+^)/H^+^ exchanger	NaCl (75 mM; 12 days)	[50]
*NHD1*	Arabidopsis	Sodium hydrogen antiporter	NaCl (150 mM; 72 h)	[51]
*KEA*	Arabidopsis	K^+^/H^+^ antiporter	NaCl (75 mM)	[52]
*YL1*	Arabidopsis	YqeH-type GTPase	NaCl (0, 150 mM; 2 days)	[53]
ABA and kinase signaling (5)				
*ABA1*	Arabidopsis	Zeaxanthin epoxidase	NaCl (300 mM; 3 h)	[54]
*NCED1*	Cowpea (*Vigna unguiculata*)	9-*cis*-epoxycarotenoid dioxygenase	NaCl (250 mM; 0, 1, 2, 5, 10, 24 h)	[55]
*MDA1*	Arabidopsis	Transcription termination factor	NaCl (100, 150, 200 mM; 4, 9, 10, 14 days)	[56]
*COR15*	Arabidopsis	15 kDa protein	NaCl (150 mM; 3 days)	[57]
*MsK4*	Alfalfa (*Medicago sativa*)	Glycogen synthase kinase 3 like kinase	NaCl (100 mM; 4 weeks)	[58]
Gene expression and protein turnover (6)				
*RH3*	Arabidopsis	DEAD-box RNA helicase	NaCl (100 mM; 0, 4, 12, 24 h)	[59]
*CRP1*	Arabidopsis	Chloroplast-targeted RNA-binding protein 1	NaCl (150 mM, 7 days)	[60]
*S-RBP11*	Arabidopsis	RNA-binding group protein	NaCl (0, 130, 140, 150, 160 mM; 14 days); (300 mM; 0, 1, 2, 4, 8 h)	[61]
*TufA*	Pea (*Pisum sativum*)	Chloroplast translation elongation factor	NaCl (100, 200, 500 mM; 4, 6, 24 h)	[62]
*PsbA*	*Synechocystis* sp. PCC 6803	Photosystem II D1 protein	NaCl (20 mM, 500 mM, 1000 mM; 0, 1, 2, 3, 4 h)	[63]
*DegP2*	Arabidopsis	Prokaryotic trypsin-type Deg/Htr serine protease	NaCl (400 mM; 2 h)	[64]

Ref.: Reference.

## Abbreviations

3-PGA	Glycerate-3-phosphate
ABA	Abscisic acid
ABC1K	Activity of bc1 complex-like kinase
ABI4	Abscisic acid insensitive 4
APX/tAPX	Ascorbate peroxidase/Thylakoid ascorbate peroxidase
AsA	Ascorbate
BADH	Betaine aldehyde dehydrogenase
CAM	Crassulacean acid metabolism
CAT	Catalase
CMO	Choline monooxygenase
CO_2_	Carbon dioxide
COR	Cold-responsive protein
Cu/Zn SOD	Copper/zinc superoxide dismutase
DegP2	Prokaryotic trypsin-type Deg/Htr serine protease
DGDG	Digalactodiacylglycerol
DHA	Dehydroascorbate
DHAR	Dehydroascorbate reductase
EF-Tu	Translation elongation factor
ETC	Electron transport chain
GAPB	Glyceraldehyde 3-phosphate dehydrogenase beta subunit
GPAT	Glycerol-3-phosphate acyltransferase
GPX	Glutathione peroxidase
GR	Glutathione reductase
GS2	Glutamine synthetase
GSH	Reduced glutathione
GSSG	Oxidized glutathione
H_2_O_2_	Hydrogen peroxide
HKT1	High-affinity K^+^ transporter 1
KEA	K^+^-efflux antiporter
MDHAR	Monodehydroascorbate reductase
Met	Methionine
MGD	Monogalactosyldiacylglycerol synthase
MGDG	Monogalactosyldiacylglycerol
MSR	Methionine sulfoxide reductase
NADP-MDH	NADP+-dependent malate dehydrogenase
NDPK2	Nucleoside diphosphate kinase 2
NTRC	NADPH thioredoxin reductase
^1^O_2_	Singlet oxygen
O_2_	Oxygen
O_2_^−^	Superoxide anion
C	Oxaloacetate
OH•	Hydroxyl radical
PS II	Photosystem II
γ-TMT	γ-tocopherol methyltransferase
RBP	RNA binding protein
RH	DEAD-box RNA helicase
ROS	Reactive oxygen species
RRM	RNA-recognition motif
RUB	Rubredoxin
Rubisco	Ribulose-1,5-bisphosphate carboxylase/oxygenase
TPP	Trehalose-6-phosphate phosphatase
Trx/Prx	Thioredoxin/Peroxiredoxin
TSPO	18 kDa translocator protein

## References

[1] Munns R., Tester M. (2008). Mechanisms of salinity tolerance. Annu. Rev. Plant Biol..

[2] Tuteja N. (2007). Chapter twenty-four-mechanisms of high salinity tolerance in plants. Methods Enzymol..

[3] Van Wijk K.J. (2000). Proteomics of the chloroplast: Experimentation and prediction. Trends Plant Sci..

[4] Baginsky S., Gruissem W. (2004). Chloroplast proteomics: Potentials and challenges. J. Exp. Bot..

[5] Abdallah F., Salamini F., Leister D. (2000). A prediction of the size and evolutionary origin of the proteome of chloroplasts of Arabidopsis. Trends Plant Sci..

[6] Arabidopsis Genome Initiative (2000). Analysis of the genome sequence of the flowering plant *Arabidopsis thaliana*. Nature.

[7] Leister D. (2003). Chloroplast research in the genomic age. Trends Genet..

[8] Peltier J.B., Emanuelsson O., Kalume D.E., Ytterberg J., Friso G., Rudella A., Liberles D.A., Söderberg L., Roepstorff P., von Heijne G. (2002). Central functions of the lumenal and peripheral thylakoid proteome of Arabidopsis determined by experimentation and genome-wide prediction. Plant Cell.

[9] Wang L., Liang W., Xing J., Tan F., Chen Y., Huang L., Cheng C.L., Chen W. (2013). Dynamics of chloroplast proteome in salt-stressed mangrove *Kandelia candel* (L.) Druce. J. Proteome Res..

[10] Fan P., Feng J., Jiang P., Chen X., Bao H., Nie L., Jiang D., Lv S., Kuang T., Li Y. (2011). Coordination of carbon fixation and nitrogen metabolism in *Salicornia europaea* under salinity: Comparative proteomic analysis on chloroplast proteins. Proteomics.

[11] Zörb C., Herbst R., Forreiter C., Schubert S. (2009). Short-term effects of salt exposure on the maize chloroplast protein pattern. Proteomics.

[12] Do Amaral M.N., Arge L.W.P., Benitez L.C., Danielowski R., da Silveira Silveira S.F., da Rosa Farias D., de Oliveira A.C., da Maia L.C., Braga E.J.B. (2016). Comparative transcriptomics of rice plants under cold, iron, and salt stresses. Funct. Integr. Genom..

[13] Edreva A. (2005). Generation and scavenging of reactive oxygen species in chloroplasts: A submolecular approach. Agr. Ecosyst. Environ..

[14] Miller G., Suzuki N., Ciftci-Yilmaz S., Mittler R. (2010). Reactive oxygen species homeostasis and signalling during drought and salinity stresses. Plant Cell Environ..

[15] Asada K. (2006). Production and scavenging of reactive oxygen species in chloroplasts and their functions. Plant Physiol..

[16] Wu J., Zhang J., Li X., Xu J., Wang L. (2016). Identification and characterization of a *PutCu/Zn-SOD* gene from *Puccinellia tenuiflora* (Turcz.) Scribn. et Merr. Plant Growth Regul..

[17] Badawi G.H., Yamauchi Y., Shimada E., Sasaki R., Kawano N., Tanaka K., Tanaka K. (2004). Enhanced tolerance to salt stress and water deficit by overexpressing superoxide dismutase in tobacco (*Nicotiana tabacum*) chloroplasts. Plant Sci..

[18] Jing X., Hou P., Lu Y., Deng S., Li N., Zhao R., Sun J., Wang Y., Han Y., Lang T. (2015). Overexpression of copper/zinc superoxide dismutase from mangrove *Kandelia candel* in tobacco enhances salinity tolerance by the reduction of reactive oxygen species in chloroplast. Front. Plant Sci..

[19] Tseng M.J., Liu C.W., Yiu J.C. (2007). Enhanced tolerance to sulfur dioxide and salt stress of transgenic Chinese cabbage plants expressing both superoxide dismutase and catalase in chloroplasts. Plant Physiol. Biochem..

[20] Luo X., Wu J., Li Y., Nan Z., Guo X., Wang Y., Zhang A., Wang Z., Xia G., Tian Y. (2013). Synergistic effects of *GhSOD1* and *GhCAT1* overexpression in cotton chloroplasts on enhancing tolerance to methyl viologen and salt stresses. PLoS ONE.

[21] Badawi G.H., Kawano N., Yamauchi Y., Shimada E., Sasaki R., Kubo A., Tanaka K. (2004). Over-expression of ascorbate peroxidase in tobacco chloroplasts enhances the tolerance to salt stress and water deficit. Physiol. Plant..

[22] Sheptovitsky Y.G., Brudvig G.W. (1996). Isolation and characterization of spinach photosystem II membrane-associated catalase and polyphenol oxidase. Biochemistry.

[23] Kavitha K., George S., Venkataraman G., Parida A. (2010). A salt-inducible chloroplastic monodehydroascorbate reductase from halophyte *Avicennia marina* confers salt stress tolerance on transgenic plants. Biochimie.

[24] Le Martret B., Poage M., Shiel K., Nugent G.D., Dix P.J. (2011). Tobacco chloroplast transformants expressing genes encoding dehydroascorbate reductase, glutathione reductase, and glutathione-*S*-transferase, exhibit altered anti-oxidant metabolism and improved abiotic stress tolerance. Plant Biotechnol. J..

[25] Wu T.M., Lin W.R., Kao Y.T., Hsu Y.T., Yeh C.H., Hong C.Y., Kao C.H. (2013). Identification and characterization of a novel chloroplast/mitochondria co-localized glutathione reductase 3 involved in salt stress response in rice. Plant Mol. Biol..

[26] Wu T.M., Lin W.R., Kao C.H., Hong C.Y. (2015). Gene knockout of glutathione reductase 3 results in increased sensitivity to salt stress in rice. Plant Mol. Boil..

[27] Jing L.W., Chen S.H., Guo X.L., Zhang H., Zhao Y.X. (2006). Overexpression of a chloroplast-located peroxiredoxin Q gene, *SsPrxQ*, increases the salt and low-temperature tolerance of Arabidopsis. J. Integr. Plant Biol..

[28] Serrato A.J., Pérez-Ruiz J.M., Spínola M.C., Cejudo F.J. (2004). A novel NADPH thioredoxin reductase, localized in the chloroplast, which deficiency causes hypersensitivity to abiotic stress in *Arabidopsis thaliana*. J. Biol. Chem..

[29] Zhai C.Z., Zhao L., Yin L.J., Chen M., Wang Q.Y., Li L.C., Xu Z.S., Ma Y.Z. (2013). Two wheat glutathione peroxidase genes whose products are located in chloroplasts improve salt and H_2_O_2_ tolerances in Arabidopsis. PLoS ONE.

[30] Yang S., Zhang Q., Li T., Du J., Yang S., Yang C. (2012). *AtSIA1*, an ABC1-like kinase, regulates salt response in Arabidopsis. Biologia.

[31] Jin S., Daniell H. (2014). Expression of γ-tocopherol methyltransferase in chloroplasts results in massive proliferation of the inner envelope membrane and decreases susceptibility to salt and metal-induced oxidative stresses by reducing reactive oxygen species. Plant Biotechnol. J..

[32] Ye W., Hu S., Wu L., Ge C., Cui Y., Chen P., Wang X., Xu J., Ren D., Dong G. (2016). *White stripe leaf 12* (*WSL12*), encoding a nucleoside diphosphate kinase 2 (OsNDPK2), regulates chloroplast development and abiotic stress response in rice (*Oryza sativa* L.). Mol. Breed..

[33] Kim Y.H., Lim S., Yang K.S., Kim C.Y., Kwon S.Y., Lee H.S., Wang X., Zhou Z., Ma D., Yun D.J. (2009). Expression of Arabidopsis *NDPK2* increases antioxidant enzyme activities and enhances tolerance to multiple environmental stresses in transgenic sweetpotato plants. Mol. Breed..

[34] Balsemão-Pires E., Jaillais Y., Olson B.J., Andrade L.R., Umen J.G., Chory J., Sachetto-Martins G. (2011). The Arabidopsis translocator protein (AtTSPO) is regulated at multiple levels in response to salt stress and perturbations in tetrapyrrole metabolism. BMC Plant Biol..

[35] Guo X., Wu Y., Wang Y., Chen Y., Chu C. (2009). OsMSRA4.1 and OsMSRB1.1, two rice plastidial methionine sulfoxide reductases, are involved in abiotic stress responses. Planta.

[36] Zhang J.T., Zhu J.Q., Zhu Q., Liu H., Gao X.S., Zhang H.X. (2009). Fatty acid desaturase-6 (Fad6) is required for salt tolerance in *Arabidopsis thaliana*. Biochem. Biophys. Res. Commun..

[37] Sun Y.L., Li F., Su N., Sun X.L., Zhao S.J., Meng Q.W. (2010). The increase in unsaturation of fatty acids of phosphatidylglycerol in thylakoid membrane enhanced salt tolerance in tomato. Photosynthetica.

[38] Wang S., Uddin M.I., Tanaka K., Yin L., Shi Z., Qi Y., Mano J., Matsui K., Shimomura N., Sakaki T. (2014). Maintenance of chloroplast structure and function by overexpression of the *OsMGD* gene leads to enhanced salt tolerance in tobacco. Plant Physiol..

[39] Li Y., Liu P., Takano T., Liu S. (2016). A chloroplast-localized rubredoxin family protein gene from *Puccinellia tenuiflora* (*PutRUB*) increases NaCl and NaHCO_3_ tolerance by decreasing H_2_O_2_ accumulation. Int. J. Mol. Sci..

[40] Khurana N., Chauhan H., Khurana P. (2015). Characterization of a chloroplast localized wheat membrane protein (TaRCI) and its role in heat, drought and salinity stress tolerance in *Arabidopsis thaliana*. Plant Gene.

[41] Cushman J.C. (1993). Molecular cloning and expression of chloroplast NADP-malate dehydrogenase during Crassulacean acid metabolism induction by salt stress. Photosynth. Res..

[42] Chang L., Guo A., Jin X., Yang Q., Wang D., Sun Y., Huang Q., Wang L., Peng C., Wang X. (2015). The β subunit of glyceraldehyde 3-phosphate dehydrogenase is an important factor for maintaining photosynthesis and plant development under salt stress-based on an integrative analysis of the structural, physiological and proteomic changes in chloroplasts in *Thellungiella halophila*. Plant Sci..

[43] Hoshida H., Tanaka Y., Hibino T., Hayashi Y., Tanaka A., Takabe T., Takabe T. (2000). Enhanced tolerance to salt stress in transgenic rice that overexpresses chloroplast glutamine synthetase. Plant Mol. Biol..

[44] Bao Y., Zhao R., Li F., Tang W., Han L. (2011). Simultaneous expression of *Spinacia oleracea* chloroplast choline monooxygenase (CMO) and betaine aldehyde dehydrogenase (BADH) genes contribute to dwarfism in transgenic *Lolium perenne*. Plant Mol. Biol. Report..

[45] Zhang J., Tan W., Yang X.H., Zhang H.X. (2008). Plastid-expressed choline monooxygenase gene improves salt and drought tolerance through accumulation of glycine betaine in tobacco. Plant Cell Rep..

[46] Kumar S., Dhingra A., Daniell H. (2004). Plastid-expressed betaine aldehyde dehydrogenase gene in carrot cultured cells, roots, and leaves confers enhanced salt tolerance. Plant Physiol..

[47] Yang X., Liang Z., Wen X., Lu C. (2008). Genetic engineering of the biosynthesis of glycinebetaine leads to increased tolerance of photosynthesis to salt stress in transgenic tobacco plants. Plant Mol. Biol..

[48] Krasensky J., Broyart C., Rabanal F.A., Jonak C. (2014). The redox-sensitive chloroplast trehalose-6-phosphate phosphatase *AtTPPD* regulates salt stress tolerance. Antioxid. Redox Signal..

[49] Patra B., Ray S., Richter A., Majumder A.L. (2010). Enhanced salt tolerance of transgenic tobacco plants by co-expression of *PcINO1* and *McIMT1* is accompanied by increased level of *myo*-inositol and methylated inositol. Protoplasma.

[50] Song C.P., Guo Y., Qiu Q., Lambert G., Galbraith D.W., Jagendorf A., Zhu J.K. (2004). A probable Na^+^(K^+^)/H^+^ exchanger on the chloroplast envelope functions in pH homeostasis and chloroplast development in *Arabidopsis thaliana*. Proc. Natl. Acad. Sci. USA.

[51] Müller M., Kunz H.H., Schroeder J.I., Kemp G., Young H.S., Neuhaus H.E. (2014). Decreased capacity for sodium export out of Arabidopsis chloroplasts impairs salt tolerance, photosynthesis and plant performance. Plant J..

[52] Kunz H.H., Gierth M., Herdean A., Satoh-Cruz M., Kramer D.M., Spetea C., Schroeder J.I. (2014). Plastidial transporters KEA1, -2, and-3 are essential for chloroplast osmoregulation, integrity, and pH regulation in Arabidopsis. Proc. Natl. Acad. Sci. USA.

[53] Li P.C., Huang J.G., Yu S.W., Li Y.Y., Sun P., Wu C.A., Zheng C.C. (2016). Arabidopsis YL1/BPG2 is involved in seedling shoot response to salt stress through ABI4. Sci. Rep..

[54] Xiong L., Lee H., Ishitani M., Zhu J.K. (2002). Regulation of osmotic stress-responsive gene expression by the *LOS6*/*ABA1* locus in Arabidopsis. J. Biol. Chem..

[55] Iuchi S., Kobayashi M., Yamaguchi-Shinozaki K., Shinozaki K. (2000). A stress-inducible gene for 9-*cis*-epoxycarotenoid dioxygenase involved in abscisic acid biosynthesis under water stress in drought-tolerant cowpea. Plant Physiol..

[56] Robles P., Micol J.L., Quesada V. (2012). Arabidopsis MDA1, a nuclear-encoded protein, functions in chloroplast development and abiotic stress responses. PLoS ONE.

[57] Liu D., Hou L., Li W.C., Cheng J.F., Fu Y.Q. (2014). *COR15B* expression is affected by chloroplast functionality and its role in response to salt stress in *Arabidopsis thaliana*. Biol. Plant..

[58] Kempa S., Rozhon W., Samaj J., Erban A., Baluška F., Becker T., Haselmayer J., Schleiff E., Kopka J., Hirt H. (2007). A plastid-localized glycogen synthase kinase 3 modulates stress tolerance and carbohydrate metabolism. Plant J..

[59] Gu L., Xu T., Lee K., Lee K.H., Kang H. (2014). A chloroplast-localized DEAD-box RNA helicase AtRH3 is essential for intron splicing and plays an important role in the growth and stress response in *Arabidopsis thaliana*. Plant Physiol. Biochem..

[60] Xu T., Sy N.D., Lee H.J., Kwak K.J., Gu L., Kim J.I., Kang H. (2014). Functional characterization of a chloroplast-targeted RNA-binding protein CRP1 in *Arabidopsis thaliana* under abiotic stress conditions. J. Plant Biol..

[61] Lee S.Y., Seok H.Y., Tarte V.N., Woo D.H., Le D.H., Lee E.H., Moon Y.H. (2014). The Arabidopsis chloroplast protein S-RBP11 is involved in oxidative and salt stress responses. Plant Cell Rep..

[62] Singh B.N., Mishra R.N., Agarwal P.K., Goswami M., Nair S., Sopory S.K., Reddy M.K. (2004). A pea chloroplast translation elongation factor that is regulated by abiotic factors. Biochem. Biophys. Res. Commun..

[63] Allakhverdiev S.I., Nishiyama Y., Miyairi S., Yamamoto H., Inagaki N., Kanesaki Y., Murata N. (2002). Salt stress inhibits the repair of photodamaged photosystem II by suppressing the transcription and translation of *psbA* genes in Synechocystis. Plant Physiol..

[64] Haußühl K., Andersson B., Adamska I. (2001). A chloroplast DegP2 protease performs the primary cleavage of the photodamaged D1 protein in plant photosystem II. EMBO J..

[65] Sevilla F., Camejo D., Ortiz-Espín A., Calderón A., Lázaro J.J., Jiménez A. (2015). The thioredoxin/peroxiredoxin/sulfiredoxin system: Current overview on its redox function in plants and regulation by reactive oxygen and nitrogen species. J. Exp. Bot..

[66] Gill S.S., Tuteja N. (2010). Reactive oxygen species and antioxidant machinery in abiotic stress tolerance in crop plants. Plant Physiol. Biochem..

[67] Martinis J., Glauser G., Valimareanu S., Kessler F. (2013). A chloroplast ABC1-like kinase regulates vitamin E metabolism in Arabidopsis. Plant Physiol..

[68] Yang K.A., Moon H., Kim G., Lim C.J., Hong J.C., Lim C.O., Yun D.J. (2003). NDP kinase 2 regulates expression of antioxidant genes in Arabidopsis. Proc. Jpn. Acad. Ser. B.

[69] Upchurch R.G. (2008). Fatty acid unsaturation, mobilization, and regulation in the response of plants to stress. Biotechnol. Lett..

[70] Loll B., Kern J., Saenger W., Zouni A., Biesiadka J. (2007). Lipids in photosystem II: Interactions with protein and cofactors. Biochim. Biophys. Acta.

[71] Lee A.G. (2000). Membrane lipids: It’s only a phase. Curr. Biol..

[72] Jordan P., Fromme P., Witt H.T., Klukas O., Saenger W., Krauß N. (2001). Three-dimensional structure of cyanobacterial photosystem I at 2.5 Å resolution. Nature.

[73] Mizusawa N., Wada H. (2012). The role of lipids in photosystem II. Biochim. Biophys. Acta.

[74] Shimojima M., Ohta H. (2011). Critical regulation of galactolipid synthesis controls membrane differentiation and remodeling in distinct plant organs and following environmental changes. Prog. Lipid Res..

[75] Kurtz D.M. (2004). Microbial detoxification of superoxide: The non-heme iron reductive paradigm for combating oxidative stress. Acc. Chem. Res..

[76] Calderon R.H., García-Cerdán J.G., Malnoë A., Cook R., Russell J.J., Gaw C., Dent R.M., de Vitry C., Niyogi K.K. (2013). A conserved rubredoxin is necessary for photosystem II accumulation in diverse oxygenic photoautotrophs. J. Biol. Chem..

[77] Majumder A.L., Sengupta S., Goswami L., Pareek A., Sopory S.K., Bohnert H.J. (2009). Osmolyte regulation in abiotic stress. Abiotic Stress Adaptation in Plants.

[78] Rhodes D., Hanson A.D. (1993). Quaternary ammonium and tertiary sulfonium compounds in higher plants. Annu. Rev. Plant Physiol. Plant Mol. Biol..

[79] Papageorgiou G.C., Murata N. (1995). The unusually strong stabilizing effects of glycine betaine on the structure and function of the oxygen-evolving photosystem II complex. Photosynth. Res..

[80] Chen T.H., Murata N. (2008). Glycinebetaine: An effective protectant against abiotic stress in plants. Trends Plant Sci..

[81] Tian F., Wang W., Liang C., Wang X., Wang G., Wang W. (2017). Overaccumulation of glycine betaine makes the function of the thylakoid membrane better in wheat under salt stress. Crop J..

[82] Berthomieu P., Conéjéro G., Nublat A., Brackenbury W.J., Lambert C., Savio C., Uozumi N., Oiki S., Yamada K., Cellier F. (2003). Functional analysis of *AtHKT1* in Arabidopsis shows that Na^+^ recirculation by the phloem is crucial for salt tolerance. EMBO J..

[83] Shkolnik-Inbar D., Adler G., Bar-Zvi D. (2013). *ABI4* downregulates expression of the sodium transporter *HKT1;1* in Arabidopsis roots and affects salt tolerance. Plant J..

[84] Nambara E., Marion-Poll A. (2005). Abscisic acid biosynthesis and catabolism. Annu. Rev. Plant Biol..

[85] Tan B.C., Joseph L.M., Deng W.T., Liu L., Li Q.B., Cline K., McCarty D.R. (2003). Molecular characterization of the Arabidopsis 9-*cis* epoxycarotenoid dioxygenase gene family. Plant J..

[86] Xu Z.Y., Kim D.H., Hwang I. (2013). ABA homeostasis and signaling involving multiple subcellular compartments and multiple receptors. Plant Cell Rep..

[87] Jung H.J., Park S.J., Kang H. (2013). Regulation of RNA metabolism in plant development and stress responses. J. Plant Biol..

[88] Rajkowitsch L., Chen D., Stampfl S., Semrad K., Waldsich C., Mayer O., Jantsch M.F., Konrat R., Bläsi U., Schroeder R. (2007). RNA chaperones, RNA annealers and RNA helicases. RNA Biol..

[89] Caldas T.D., El Yaagoubi A., Richarme G. (1998). Chaperone properties of bacterial elongation factor EF-Tu. J. Biol. Chem..

[90] Takahashi S., Badger M.R. (2011). Photoprotection in plants: A new light on photosystem II damage. Trends Plant Sci..

[91] Nixon P.J., Barker M., Boehm M., de Vries R., Komenda J. (2005). FtsH-mediated repair of the photosystem II complex in response to light stress. J. Exp. Bot..

[92] Fan P., Nie L., Jiang P., Feng J., Lv S., Chen X., Bao H., Guo J., Tai F., Wang J. (2013). Transcriptome analysis of *Salicornia europaea* under saline conditions revealed the adaptive primary metabolic pathways as early events to facilitate salt adaptation. PLoS ONE.

